# SERIES: eHealth in primary care. Part 5: A critical appraisal of five widely used eHealth applications for primary care – opportunities and challenges

**DOI:** 10.1080/13814788.2021.1962845

**Published:** 2021-08-25

**Authors:** Marise J. Kasteleyn, Anke Versluis, Petra van Peet, Ulrik Bak Kirk, Jens van Dalfsen, Eline Meijer, Persijn Honkoop, Kendall Ho, Niels H. Chavannes, Esther P. W. A. Talboom-Kamp

**Affiliations:** a Department of Public Health and Primary Care (PHEG), Leiden University Medical Centre, Leiden, The Netherlands; b National eHealth Living Lab (NeLL), Leiden, The Netherlands; c The Research Unit for General Practice, Aarhus, Denmark; d The European Society for Quality and Safety in Family Practice (EQuiP), Aarhus, Denmark; e Faculty of Medicine, eHealth Strategy Office, University of British Columbia, Vancouver, Canada; f Saltro Diagnostic Center, Utrecht, The Netherlands

**Keywords:** eHealth, primary care, challenges, opportunities

## Abstract

**Background:**

Given the pressure on modern healthcare systems, eHealth can offer valuable opportunities. However, understanding the potential and challenges of eHealth in daily practice can be challenging for many general practitioners (GPs) and their staff.

**Objectives:**

To critically appraise five widely used eHealth applications, in relation to safe, evidence-based and high-quality eHealth. Using these applications as examples, we aim to increase understanding of eHealth among GPs and highlight the opportunities and challenges presented by eHealth.

**Discussion:**

eHealth applications can support patients while increasing efficiency for GPs. A three-way division (*inform, monitor, track; interaction; data utilisation*) characterises many eHealth applications, with an increasing degree of complexity depending on the domain. All applications provide information and some have extra functionalities that promote interaction, while data analysis and artificial intelligence may be applied to support or (fully) automate care processes. Applications in the inform domain are relatively easy to use and implement but their impact on clinical outcomes may be limited. More demanding applications, in terms of privacy and ethical aspects, are found in the data utilisation domain and may potentially have a more significant impact on care processes and patient outcomes. When selecting and implementing eHealth applications, we recommend that GPs remain critical regarding preconditions on safe, evidence-based and high-quality eHealth, particularly in the case of more complex applications in the data utilisation domain.

KEY MESSAGES

- eHealth applications show varying degrees of complexity; while all applications generally provide information, additional features may support interaction and in advanced applications data analysis can automate processes.
- High-risk and high-gain: the higher the complexity, the higher the potential impact.
- Scientific evidence on effectiveness is often lacking or of insufficient quality.

## Introduction

### eHealth in primary care

Healthcare worldwide faces a heavier and increasingly complex workload due to an ageing population and a rise in multimorbidity [1]. Primary care bears the full brunt of this pressure because it is usually the first port-of-call for the general population [2]. This increasing pressure creates a necessity for a more efficient organisation and better distribution of care [3]. Inherent to this change are general practitioners (GPs) and their staff who have to deal with changing patient-provider relationships. Tailored care that focuses on a patient’s autonomy, self-management and self-efficacy is becoming increasingly important [4]. The COVID-19 pandemic further underlines the need for a transformation of care, as continuity of care should be secured even in times of limited access to face-to-face care [5].

**Figure 1.**
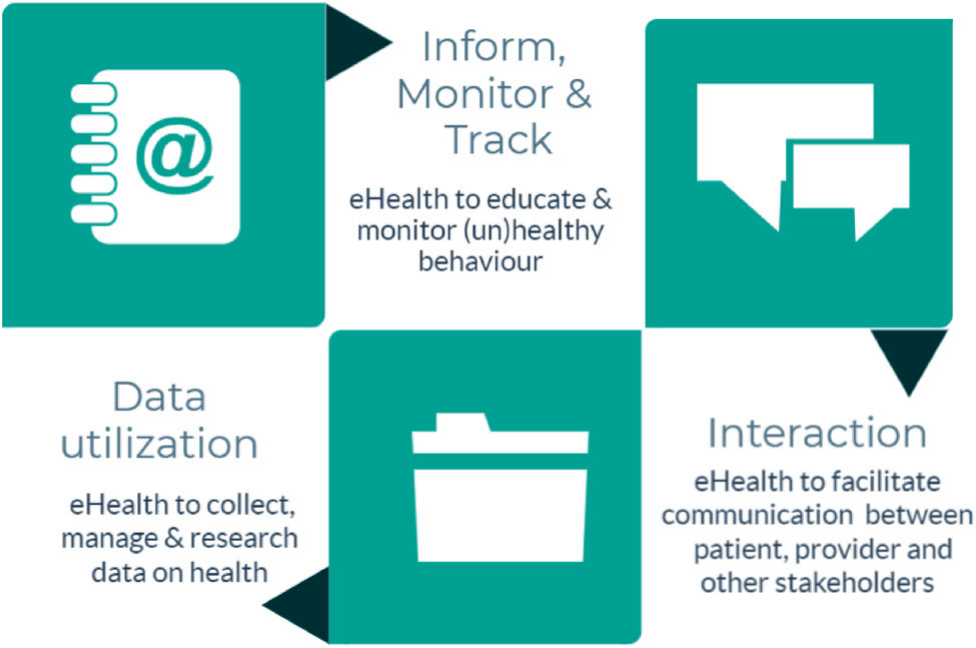
Conceptualisation of eHealth in primary care, derived from Shaw et al. [11].

eHealth has been proposed as a valuable tool to support healthcare and ensure universal health coverage [1]. According to Shaw et al. (Figure 1) [6], eHealth has three main functions: (1) ‘Inform, monitor and track’ to observe and study health parameters; (2) ‘Interaction’ to support communication; and (3) ‘Data utilisation’ to collect, manage, and use health data [1]. In daily practice, eHealth applications ranging from mobile phone applications to telemonitoring systems often encompass multiple domains.

In the first four articles of this series on eHealth, we addressed issues related to the successful uptake and use of eHealth in primary care [1,7–9]. These issues were described from various perspectives ranging from ethical aspects to education and implementation, including the six preconditions that we view as pivotal to ensure safe, evidence-based and high-quality eHealth (Box 1). However, for many GPs, it may still be challenging to maintain an up-to-date overview of the opportunities and challenges pertaining to the use of eHealth in daily practice. Therefore, this opinion paper aims to apply the six preconditions for high-quality eHealth, which are described in detail in Part 1 of the SERIES [1], to critically appraise specific and tangible eHealth applications currently in use in primary care. We intend to increase GPs’ understanding of eHealth and provide insight into the opportunities and challenges concerning the use of eHealth. Using examples illustrating the potential of eHealth, we hope to inspire and encourage GPs, while at the same time remaining reflective and critical regarding eHealth applications in daily practice.

## Contextual factors

### Country-specific aspects

Adoption of eHealth differs among and within countries and is influenced by several factors, such as the details of national policy. Regarding the six preconditions (co-creation, blended care, individualisation, globalisation, evidence and ethics), there is a lack of consistent national monitoring or thorough evaluation of eHealth internationally [10]. In existing literature, several countries are mentioned repeatedly because of well-established digital health infrastructure. Denmark is considered to be an international front runner because of the combination of an advanced high-quality digital health system and an excellent nationwide Digital Health Strategy that focuses on co-creation, blended care, individualisation and ethics [11]. A comprehensive digital system enables Danish GPs, healthcare facilities, nursing homes and other facilities to cooperate better and decrease inequalities. In Australia, a national digital strategy focuses on leveraging co-creation and blended care in a digitally-savvy society, combined with a clear digital health vision that places the individual patient at the centre of care [12].

Several countries, including New Zealand, the Nordic countries and Israel, are relatively advanced in adopting eHealth and have a sound digital strategy in place that details preconditions for co-creation and blended care [12]. In New Zealand, regional systems integrate data from several primary care providers (i.e. community nurses, pharmacists and GPs) and hospitals, forming a broadly accessible data structure (depending on access rights) [13]. In the Netherlands, most primary care practices offer eHealth, although the level of use varies considerably and co-creation with the patient is still rare [14]. In Canada and the United States, GPs are increasingly adopting eHealth, including telemedicine for online consultation, a trend further boosted by COVID-19 [15]. The challenge now is to find a responsible approach to maintaining the use of eHealth as the COVID-19 pandemic recedes.

### General practitioner-specific aspects

The speed of eHealth implementation depends for a large part on the willingness of physicians. While some healthcare providers can be considered early adopters and are open to using eHealth in daily practice [16], others may be more reluctant to change their routines. The expectations of general practitioners concerning the impact of eHealth on workload are sometimes conflicting, with one qualitative study stating that the majority of general practitioners thought that eHealth would reduce their workload while another large survey in the same country found that only 10% had similar expectations [17,18].

Other factors influencing the use and adoption of eHealth in daily practice relate to healthcare providers’ preferences, e.g. seeing patients face-to-face and perceptions about the (added) value of e-consultations or telemonitoring in relation to improving care and relationships with patients [19]. Legislative aspects may also play a role, as there can be uncertainty about professional responsibilities [7]. Furthermore, healthcare providers appear less inclined to use eHealth if they are worried about their job security or cost aspects, citing issues such as the extra costs of eHealth applications without compensation or the loss of revenue if the costs but not the benefits are felt at the practice level [19,20]. Differing preferences may also be linked to a lack of knowledge about and attitudes towards eHealth’s opportunities and challenges.

### Aspects concerning the complexity of eHealth applications

The complexity of an eHealth application, ranging from less complex applications in the inform domain to more complex applications in the data utilisation domain, together with the required level of care reorganisation, directly affects adoption rates [20]. Using a relatively simple eHealth application to replace a specific routine, e.g. tracking the lifestyles of patients with chronic diseases with an app rather than a paper diary, requires different abilities compared to applications that radically change work processes, e.g. 24/7 telemonitoring of patients with diabetes rather than seeing them on a quarterly basis. The lessons learned from the first four papers in the eHealth series are relevant and important to all applications, especially in the case of more complex applications [1,7–9]. Furthermore, patient factors are related to the complexity of applications and need to be considered. For example, a fair degree of (health) literacy and digital competencies are required, especially for complex applications [1]. Other factors influencing the use of eHealth include age, income and education [21]. Iterative co-creation processes with relevant target groups are essential during the development and implementation of eHealth applications.

## eHealth applications

To support GPs interested in adopting eHealth, we describe five widely-used eHealth applications. Applications were eligible for inclusion in the paper if they were relevant to primary care and currently in wide use. As it was not our aim to provide a comprehensive overview of all existing applications, we selected only five applications. To cover all the domains detailed by Shaw et al., we set at least one application per domain, with the applications serving as examples to illustrate the opportunities and challenges of eHealth in primary care and as aids to help define critical aspects of concern to GPs. Using these examples, we elaborate on the preconditions for safe, evidence-based and high-quality eHealth (Box 1) and the five examples highlight how these preconditions ultimately influence the adoption and utilisation of eHealth applications.

The five included applications were selected pragmatically, based on consensus and brainstorm sessions with the co-authors. One of the authors (ET) checked the literature, websites and the application itself to extract information regarding the six preconditions for all applications. MK also reviewed the literature, websites and applications to validate the results. The next step was validation of the results by the whole author group and discussion to form the final opinion and interpretation of the findings.

The tables presented below summarise the five commonly used eHealth applications. Table 1 provides a description of the relevant domain(s) in relation to the Shaw classification. Table 2 describes preconditions for safe, evidence-based and high-quality eHealth.

**Table 1.** Description of five widely used eHealth applications.

Description	Shaw domain
Thuisarts.nl	
https://www.thuisarts.nl. Non-commercial public website in the Netherlands, launched by the Dutch College of General Practitioners. It provides patients with understandable and reliable medical information combined with automated (non-personal) advice. It includes the content of evidence-based guidelines on 600 topics. Each topic consists of several ‘patient situations’ (e.g. I have the flu, my child has the flu) and provides information on what to do and when to contact the GP. It includes illustrations, short videos, patient decision aids and eHealth self-management tools. It has an extremely high in-country use (24 million visitors in 2018) [37]. https://gpinfo.nl is the English version of Thuisarts.nl, covering a selection of medical topics.	Inform, monitor, track
Liva Healthcare	
https://livahealthcare.com. Innovative digital health programs to improve a variety of lifestyle behaviours by creating bonds between the lifestyle coach and the patient. The digital behavioural change programs consist of real-time personal coaching and pre-recorded videos, group-based interventions, tailored health plans, goal-tracking and self-monitoring, and fixed evidence-based curriculums personalised to support people at risk of or living with chronic conditions. Developed in Denmark in 2015 and was recently implemented in Norway, UK and Australia.	Inform, monitor, track Interaction
SHUTi	
https://www.somryst.com. An online intervention for the treatment of insomnia in adults. It is a fully automated, interactive and tailored web-based program that incorporates the primary tenets of face-to-face Cognitive Behavioural Therapy for Insomnia (CBT-I), including sleep restriction, stimulus control, cognitive restructuring, sleep hygiene and relapse prevention. Developed in the United States.	Inform, monitor, track Interaction
Babylon	
https://www.babylonhealth.com. AI system that can receive data about a patient’s symptoms, compare the information to a database of known conditions and illnesses, and then identify a course of action and related risk factors. It contains a virtual GP appointment, digital prescriptions, digital health check, instant symptom chequer, online view of medical records, and a chatbot. It is now an integrated part of the UK-based NHS. It can be used by patients who have registered for the service. Patients register with ‘GP at Hand’ and can then consult a digital GP within minutes of registration; however, they have to switch from their current GP practice to Babylon. Certain GPs work specifically for Babylon, with whom the patient registers. Video appointments are possible 24/7 in collaboration with 7 clinics in London and 1 clinic in Birmingham.	Inform, monitor, track Interaction Data utilisation
SkinVision	
https://www.skinvision.com/nl/. International skin cancer detection app allows patients to check suspicious skin spots themselves, promoting timely, appropriate care. It is available as either a freely downloadable app or a paid service, which various health insurers reimburse. A user can self-assess the skin cancer risk of a skin lesion by taking a photo with his/her smartphone, which is then processed by an algorithm. The user receives feedback on the risk presented by the skin lesion. SkinVision was developed by an official dermatology clinic regulated by the Dutch healthcare system, and can replace a normal dermatology consultation. Patients can also choose to send the photo to their healthcare professional, who will also receive the algorithm’s outcome as support in their diagnosis. The app also has a tracking function that can track skin spots over time. https://www.healthnavigator.org.nz/apps/f/firstcheck-app/. is a similar version from New Zealand	Inform, monitor, track Interaction Data utilisation

AI: Artificial Intelligence; CBT: cognitive behavioural therapy; GP: general practitioner; NHS: National Health Service; UK: United Kingdom.

**Table 2.** Evaluation of five widely used eHealth applications using six preconditions for high-quality eHealth.

Co-creation	Blended care	Individual and inclusive Applicable in high and low resource setting	Scientific evaluation	Ethics	Summary and recommendations
Thuisarts.nl					
Team of physicians and editors developed the content End-users involved in a later phase Process of co-creation could be improved	Intended use of Thuisarts.nl: by the general public/patients; to obtain information about their symptoms or before a consultation GP; to explain medical information GP; refer a patient to the website GP; to check if their advice is up to date As patients have a better understanding of their complaints, the consultation could be more tailored.	Information is simply phrased, and supported by pictures and videos The popularity of the website has grown exponentially Several million unique page views each month Also used by an older population [29]	After launch, a decreasing trend in consultation rates, mainly caused by a decrease in short- and telephone consultations [29] No evidence available on usability or impact on patient outcomes	No known adverse or unintended effects among the patient population Further improvement could be made by adding an ethical expert to the team, and by promoting co-creation with vulnerable and low-literacy patient groups	Website that informs patients and GP’s about basic medical conditions. Simple application, without a major negative impact on daily routines. However, Thuisarts.nl has not yet systematically adapted to the wishes and requirements of the end-users, which we believe is a precondition for the development of a professional and high-quality eHealth application
Liva Healthcare					
Development by: entrepreneurs behind NetDoctor.com multidisciplinary teams of healthcare professionals, software developers, scientists and patients	The healthcare professional refers a patient to Liva Healthcare No direct consequences for the routines of the referring healthcare professional An important driver of long-term weight loss was a strong relationship with a personal coach	More accessible than conventional treatment and ‘easy to use’ [30] Participants felt that having an initial one-off, face-to-face meeting with the dietician or lifestyle coach was important for their future web-based interaction	Effective in improving the lifestyle of patients with diabetes, e.g. more exercise and weight loss [32,33] Open to peer-reviewed scientific evaluation; programme will be improved after analysis by scientists [30]	No adverse or unintended effects among the patient population Further improvement could be made by adding an ethical expert to the team, and by promoting co-creation with vulnerable and low-literacy patient groups	Liva Healthcare is an easy-to-use application to improve lifestyle, with evidence-based results on clinical outcomes, high user-satisfaction and limited resistance from doctors [38]. This eHealth tool can support behavioural changes through monitoring and providing relevant feedback. Support from family and peers also matters, and long-term success depends on ability to establish positive day-to-day support [30]
SHUTi					
Development by multidisciplinary team of: psychologists psychiatrist engineers software developers commercial experts	GP’s can prescribe the SHUTi program for treatment of insomnia in adults The acceptance of this online cognitive behavioural therapy is adequate, as GP’s generally recognise the value for the treatment of insomnia. No frequent referral because of consultation time constraints and limited knowledge [39] No specific education of healthcare professionals is needed, and elaborate implementation strategies are not necessary as healthcare professionals can simply prescribe the online programme [40]	Acceptance by patients was high, with a relatively high usage and adherence [34] The design of SHUTi aims to simulate the schedule and framework of an in-person treatment. It uses approachable and supportive language to help the user feel encouraged and motivated [41]	The efficacy of SHUTi has been demonstrated in multiple randomised controlled trials, which reported a significant and persistent reduction in symptoms of insomnia [34,42] and depression [42] A recent systematic review and meta-analysis confirms the general efficacy of similar online programs [43] A subsequent version of SHUTi (i.e. Somryst) recently obtained authorisation from the FDA in the USA to be used as a ‘prescription digital therapeutic’ for the treatment of insomnia in adult patients	No adverse or unintended effects among the patient population Further improvement could be made by adding an ethical expert to the team, and by promoting co-creation with vulnerable and low-literacy patient groups	Effective and practical online solution for the treatment of insomnia in general practice. Insomnia is currently not adequately treated in the primary care setting [44] due to limited knowledge of sleep treatments [39] . Hence, contrary to international clinical guidelines that recommend CBT-I as the first line treatment for insomnia, hypnotic medication remains the primary treatment modality in the majority of the patients [45]. SHUTi is considered a *via*ble option in the treatment of insomnia [43]
Babylon					
Development by: medical doctors scientists engineers working for NHS and Babylon Babylon is regulated by the Care Quality Commission in England	Using Babylon means a different way of working for GP’s, with a more coaching, supportive role Patients do not need to learn a lot; in practice, a lot of young and healthy patients use this service	The advantages are: 24/7 digital access high satisfaction by users support by the government	No analysis by an external academic partner Impossible to determine how well it would perform with a broader set of cases Babylon's own study does not offer convincing evidence that its system can outperform GPs in any realistic situation, and there is a possibility that it might perform significantly worse when diagnosing disease [46]	To convince citizens to use Babylon Health, the developers use highly developed marketing techniques Data are stored to promote development of algorithms, without transparency in the algorithm method. Further clinical evaluation is necessary to ensure confidence in patient safety, privacy and accuracy of the algorithms [46] Possible risk that it improves the health status of the so-called ‘worried well’, but not that of vulnerable, high-risk groups	AI-based platform that claims to help patients to make a diagnosis. The usability is good, but there is no peer-reviewed analysis of data and a significant risk of underperforming diagnostics
SkinVision					
Development by a multidisciplinary team: dermatologists scientists engineers According to existing law SkinVision is a healthcare provider and must meet established qualitative and safety requirements. Initially, it did not comply with legal requirements and, among others, had to draw up a policy plan by October 2020 [47]	False-negative outcomes might delay the detection and treatment of skin cancer False-positive outcomes may cause unnecessary stress among users or unnecessary visits to GP’s The user may ignore the advice given in the smartphone app due to a lack of trust or unawareness GP’s need to understand the basics of AI to deal with such algorithms in the diagnostic process	The app seems easy to use. Users need to be able to take a photo with their mobile phones There are no (scientific) publications available on usability	The accuracy of algorithm-based smartphone apps for the assessment of skin cancer is debateable [35] The algorithm has a sensitivity of 95% and a specificity of 78.3% in recognising signs of skin cancer [36] SkinVision outperforms other skin cancer detection apps in terms of accuracy [35] The current accuracy of the algorithm may warrant its use by lay users or GP’s	The medical consequences of the use of SkinVision are still unclear Although studies have demonstrated that SkinVision can have beneficial effects when diagnosing skin cancer, adverse effects have also been reported [35] Patient safety is not fully guaranteed in this phase	Skin cancer detection app for patients and doctors, based on an algorithm with high sensitivity and moderate specificity to detect skin cancer; there is room for improvement in terms of specificity and in terms of qualitative and safety requirements

AI: Artificial intelligence; GP: general practitioner; QoL: quality of life.

Box 1 Conditions to ensure safe, evidence-based and high-quality eHealth [2]

**Table:** 

Engagement of and co-creation by all stakeholders Blended care: eHealth combined with regular care Individualised and inclusive Applicable in high- and low-resource settings Evidence-based and supported by educational guidance Being attentive to ethical considerations, privacy and patient safety

## Discussion

### Main findings

The goal of this opinion paper was to provide insight into and give examples of the opportunities and challenges pertaining to the use of eHealth in primary care. We will discuss five specific and tangible eHealth applications currently used in primary care. Our critical appraisal of these applications confirmed the three-way division of eHealth applications, characterised by increasing degrees of complexity. While all five applications provide information, interaction can be added to expand functionalities (Liva Healthcare and SHUTi), and data analysis and artificial intelligence can optionally be used to (fully) automate care processes (Babylon and SkinVision). Preconditions on safe and high-quality eHealth are relevant for all applications. Still, following our evaluation, we now argue that this is especially the case for more complex interventions in the data utilisation domain. In our opinion, these applications possess high-risk, high-gain potential both at the patient and GP level. To clarify, applications in the information domain, such as Thuisarts.nl, as well as some in the interaction domain, such as Liva Healthcare, can be readily recommended by any GP. Risks due to privacy or ethical issues are minimal, as no or limited data are collected and no drastic changes are made to the clinical process. While the impact on clinical outcomes of applications in the information domain may be minimal, the impact of applications in the interaction domain is likely to be much more profound. Applications in the data utilisation domain present significantly greater risk, as ethical aspects including patient safety and privacy might be at stake if an algorithm is inaccurate or data are not safely stored. On the other hand, the potential yield regarding patient outcomes and care processes is often high, e.g. diagnostic processes are performed at home instead of in a clinical setting, thereby increasing efficiency, or algorithms are used that outperform doctors in, for example, diagnostic processes and thus positively affect patient outcomes.

### Impact on the care process

While patients seem very satisfied with applications that automatically use data, GP resistance tends to increase when they feel that their control of the care process is diminishing or if there is uncertainty surrounding the reliability and security of data [22,23]. Nevertheless, it is doubtful that all patients fully understand the risks of these types of applications and initial satisfaction will probably diminish following negative experiences, such as when an app ‘gets it wrong’ or after a data leak. If using an eHealth application is too disruptive to regular GP routines or if it leads to higher costs, job insecurity or loss of revenue, GPs will understandably perceive this as a barrier to using the application [19]. Moreover, the level of proficiency in the use of information technologies might also affect the use of eHealth [8]. Applications that mainly provide information to patients, such as *Thuisarts.nl*, are the least ‘threatening’ for GPs, offer multiple ways to support their care processes and understandably are also popular with GPs. Applications like *SkinVision* could radically change the diagnostic process, require GPs to have a basic understanding of AI and therefore have a greater impact on daily GP practice. Clearly, the degree of disruption to a GP practice will form a barrier to using an eHealth application [9]. The greater the disruption, the greater the need for reliable testing of technical accuracy and clinical effectiveness. GPs should also consider that implementing complex eHealth interventions might require the reorganisation of care [24]. If adopted, eHealth applications must be implemented and integrated into the daily work processes of GPs.

### Scientific evaluation

While scientific evaluation of eHealth applications is essential to ensuring the safety and successful implementation of eHealth [25], scientific evidence concerning effectiveness, usability and accuracy is often lacking [26,27] or is of insufficient quality [28]. This is a particularly thorny issue in the data utilisation domain, where scientific evaluation is still in an early phase, meaning that critical external scientific evaluation is an ongoing process. The applications discussed here differ greatly in terms of supporting evidence on efficacy. Evidence concerning *thuisarts.nl* is available, but this focuses solely on healthcare usage [29], and no peer-reviewed evidence is available regarding, for example, usability or outcomes. *Liva Healthcare* and *SHUTi* have undergone more extensive testing, including their effects on patient outcomes and experiences [30–34]. Even though this evidence may be derived from the general population, this information is still relevant to primary care. Nonetheless, further investigation in a primary care setting would be valuable. Evidence is available regarding the accuracy and performance of *SkinVision* [35,36], but additional real-life studies are needed. *Babylon* is claimed to be effective, but there is no evidence to support this. Furthermore, it should be noted that (co)founders or employers of *Thuisarts.nl, Liva Healthcare* and *SkinVision* are co-authors of the scientific publications describing these applications. We suggest GPs remain critical towards eHealth applications in terms of available evidence and to check whether demonstrably independent experts provide effectiveness.

## Take home messages

The main advantages of the applications described in this paper, considerations that often apply to eHealth in general, are the relative ease of use for many patients, the high level of satisfaction, and the higher degree of independence and 24/7 applicability. In parallel, GPs can work more efficiently because eHealth supports patient self-management, lowering the demand for physical consultations. Nevertheless, perceived barriers for large-scale implementation include dealing with complex applications or a feeling of diminishing control of the clinical process.

In Part 1 of the SERIES, we concluded that scientific research on eHealth applications is essential, preferably performed continuously while incorporating different viewpoints [1]. With the current paper, it became clear that evidence is often limited, even in already widely-used applications. Although some research is available on the applications included here, it mainly focussed on one specific aspect and continuous assessment of effectiveness was lacking. Furthermore, some studies were performed by the developers rather than by independent scientists, which might have compromised reliability. Therefore, we recommend a critical stance regarding eHealth applications, which should include assessing any external scientific evaluation that may have been performed. Independent institutes could play a leading role in collecting and publishing this evidence. If eHealth applications are not validated by peer-reviewed scientific research, they should be implemented only with great restraint. If an application is likely to cause disruption to a GP’s routine work processes, such as the case with *Babylon*, we believe that effectiveness must be proven before implementation.

A limitation of this paper was the description of only five selected applications, as we wished to discuss the potential of eHealth based on specific examples. To obtain a broader overview of the availability and effectiveness of eHealth in primary care, it might be interesting to perform an umbrella review summarising published systematic reviews in this field.

## Conclusion

eHealth applications in primary care appear to exhibit a three-way division, with increasing degrees of complexity ranging from relatively simple applications providing information to more complex applications that use data and/or change the organisation of care. To select viable eHealth applications for implementation in primary care, six preconditions that ensure safe, evidence-based and high-quality eHealth must be met, particularly in the case of more complex applications.
